# Correlations of IL-17 and NF-κB gene polymorphisms with susceptibility and prognosis in acute respiratory distress syndrome in a chinese population

**DOI:** 10.1042/BSR20181987

**Published:** 2019-02-08

**Authors:** Meimei Xie, Bihuan Cheng, Yueping Ding, Changliang Wang, Jianshi Chen

**Affiliations:** 1 Department of Intensive Care Unit, the Second Affiliated Hospital and Yuying Children’s Hospital of Wenzhou Medical University, Wenzhou, P.R. China; 2 Department of Intensive Care Unit, the Second Affiliated Hospital of Zhejiang Chinese Medical University, Hangzhou, P.R. China; 3 Department of Intensive Care Unit, Sir Run Run Shaw Hospital, Zhejiang University School of Medicine, Hangzhou, P.R. China

**Keywords:** acute respiratory distress syndrome, IL-17, NF-κB1, single nucleotide polymorphism

## Abstract

The present study was performed to investigate the association between interleukin-17 (IL-17) and nuclear factor κB (NF-κB) gene polymorphisms and the risk and prognosis of acute respiratory distress syndrome (ARDS) in a Chinese population. A total of 210 Chinese patients with ARDS were selected as the study group, 210 individuals who were identified as at-risk patients but did not meet criteria for ARDS were recruited as the control group. Three single nucleotide polymorphisms (SNPs) of IL-17, including rs763780 (A>G), rs2275913 (G>A), rs8193036 (C>T) and NF-κB1 gene rs3774934 (G>A) loci were examined by Sanger sequencing technique in the peripheral blood of all subjects. Patients were followed for 30-day survival. The IL-17 rs763780 and NF-κB1 rs3774934 SNPs had no impact on ARDS risk and prognosis of ARDS (*P*>0.05). Compared with individuals carrying the wild-type GG genotype of rs2275913 at IL-17, the AA-homozygous and GA- heterozygous individuals were protected from the development of ARDS. Consistently, a decreased 30-day mortality risk was found among A-allele carriers of rs2275913 at IL-17 (*p*<0.05). For IL-17 rs8193036 SNP, the homozygote TT genotype and heterozygote CT genotypes were associated with increased ARDS susceptibility and 30-day mortality risk (*P*<0.05). Besides, decreased IL-17 levels were found in A-allele carriers of IL-17 rs2275913, whereas individuals carrying T-allele of IL-17 rs8193036 were found to have significantly increased levels of IL-17 (*P*<0.05). Our results suggested that two functional polymorphisms of IL-17, rs2275913 and rs8193036 were associated with ARDS risk and prognosis, indicating that the two genetic variants might act as possible markers for the prediction of ARDS risk and development.

## Introduction

Acute respiratory distress syndrome (ARDS) is a devastating clinical syndrome which is most commonly a manifestation of sepsis-induced organ dysfunction, characterized by disruption of endothelial barrier integrity and diffuse lung damage [1]. This can result in increased vascular permeability of alveolar-capillary membrane, acute onset of pulmonary edema, along with bilateral pulmonary infiltrates and decreased lung compliance that patients with ARDS need supportive care in the intensive care unit (ICU) to maintain oxygenation and prevent adverse outcomes [2,3]. Despite various therapeutic strategies, the mortality of ARDS remain as high as 40%. It is clinically imperative to clarify the pathophysiology of the syndrome and to identify markers that predict the risk and development of the disease.

Recent evidence show that activated leukocytes and cytokines play crucial roles in the pathogenesis of ARDS [4,5]. Interleukin-17 (IL-17) is a proinflammatory cytokine produced by the memory CD4 + T cells after activation and has been shown to be involved in amplifying inflammatory response by recruiting immune cells such as neutrophils and monocytes and inducing other proinflammatory molecules [6]. Up-regulation of IL-17 expression has been shown to correlate with the development of severe acute lung injury (ALI) in lipopolysaccharide-induced mouse model [7,8]. Increased IL-17 was found in patients with sepsis-induced ARDS and IL-17 may serve as a biomarker to indicate the severity of ARDS. Nuclear factor κB (NF-κB) is a transcription factor that regulates the expression of many cytokines and has been shown to be involved in the pathogenesis of many inflammatory disease, including ALI and ARDS. Activation and NF-κB has been shown to be a critical step in the initiation of inflammatory reaction and reduced activity of NF-κB pathway can lead to milder ALI [9,10].

Uncontrolled inflammation has been widely accepted as a hallmarker of ARDS; however, the regulation of genes within the inflammatory pathways is not well understood yet, any changes in structure and expression, may be due to genetic variations, might affect cytokine production and ARDS development. A common type of genetic variation in the genome is single nucleotide polymorphism (SNP). IL-17 have many SNPs, among which rs763780 (A>G), rs2275913 (G>A) and rs8193036 (C>T) loci are located in 6p12.1 chromosome and have been shown to correlate with aggressive disease and poorer survival in recent series with inflammatory disease [11]. NF-κB1 rs3774934 (G>A) is located in the intro region, whereas this polymorphism has been found to be associated with increased risk of epithelial ovarian cancer in a Chinese population [12]. However, whether genetic variation with these loci contributes to ARDS risk and outcome is unknown. Therefore, SNPs of these loci were selected for examination in this case–control study with the aim of unmasking the regulatory mechanisms IL-17 and NF-κB1 production and providing a genetic marker to predict ARDS risk and development.

## Materials and methods

### Patients’ characteristics

From May 2014 to October 2017, a total of 210 consecutive patients with ARDS admitted to our intensive care unit (ICU) at the Second Affiliated Hospital and Yuying Children’s Hospital of Wenzhou Medical University were enrolled in the present study as the case group, 210 controls were enrolled from individuals who were identified as at-risk patients who did not meet criteria for ARDS during the ICU stay and had no prior history of ARDS. The present study was approved by the ethics committee of the Second Affiliated Hospital and Yuying Children’s Hospital of Wenzhou Medical University. The diagnosis of ARDS was defined according to the Berlin Definition [13]. Patients were excluded if they met with the following: (1) had history of ARDS; (2) received the treatment of angiotensin-converting enzyme inhibitors or angiotensin receptor blockers one month before the onset of ARDS; (3) required medical ventilation due to chronic respiratory failure. Written, informed consents were obtained from all the patients (or their legal representatives) enrolled in the present study.

### Genotyping of IL-17 and NF-κB1 gene

**Figure 1:**
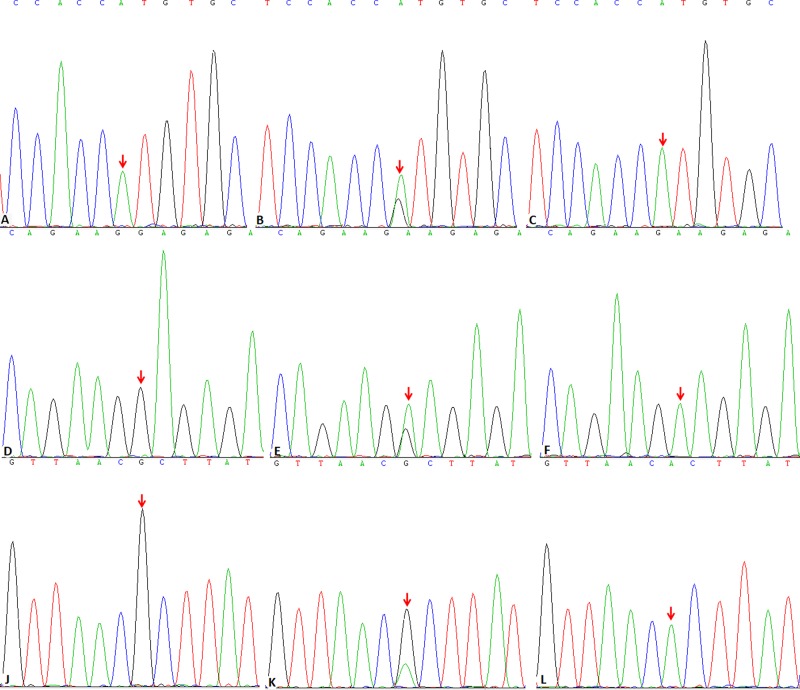
Sequencing of IL-17 and NF-κB1 SNP loci (**A**-**C**): IL-17 rs763780 (7488 A/G) AA, AG and GG genotypes; (**D**-**F**): IL-17 rs2275913 (-197 G/A) GG, GA and AA genotypes; (**G**-**I**): IL-17 rs8193036 (-737 C/T) CC, CT and TT genotypes; (**J**-**L**): NF-κB1 rs3774934 (4531 G/A) GG, GA and AA genotypes.

Each participant donated a 5-ml blood sample from ulnar vein. DNA were extracted from 2-ml blood of each sample using QIAamp DSP DNA Blood Mini Kit (Qiagen, German) and then stored at −80°C. The remaining blood was centrifuged, serum separated (3000 rpm, 15min) and stored at −80°C. Four SNPs: rs763780, rs2275913, rs8193036 of IL-17 and rs3774934 of one NF-κB1 were detected by Sanger sequencing method. The sequences of polymerase chain reaction (PCR) amplification primer were shown in Table 1. The PCR reaction system was 50 μl, including 5 μl of 10× buffer, 1 μl of 10 pmol upstream and 1 μl of 10 pmol downstream primer, 3 μL of Mg^2+^, 1 μl of dNTP, 0.4 μl of Taq enzyme, 10 ng of gDNA and water. The procedure of PCR: initialization through a temperature of 95°C for 3 min; denaturation through a temperature of 95°C for 3 s; annealing through a temperature of 60°C for 30 s; extension through a temperature of 72°C for 30 s with 35 cycles; final elongation through a temperature of 72°C for 2 min; final hold cools the reaction chamber to 4°C for an indefinite time. The production of PCR was purified using QIAquick PCR purification Kit (Qiagen, German). Then the focused segments were examined using Sanger sequencing (Figure 1).

**Table 1:** Amplification primers and PCR amplification product information

Gene	SNP	Primer sequences	Annealing temperature (°C)
IL-17	rs763780	Forward: ACCCCTGTCATCCACCATGT	60
	(7488 A/G)	Reverse: GTGGCATTTCTACAGCTTCTTCA	
	rs2275913	Forward: CTATGACCTCATTGGGGGCG	60
	(-197 G/A)	Reverse: GGTCACTTACGTGGCGTGTC	
	rs8193036	Forward: CCATCTCCATCACCTTTGTCCA	60
	(-737 C/T)	Reverse: TTCCAACCCTGCATGCTACC	
NF-κB1	rs3774934	Forward:TTTCTGTACACTCCATTGAAACTGT	60
	(4531 G/A)	Reverse: GCAGTGCTTGTAGCATTGTTTCT	

### Testing for IL-17 and NF-κB in serum

The serum samples were warmed to room temperature (20–28°C) and then detected using dedicated Enzyme linked immunosorbent assay (ELISA) kits (IL-17 Cat No. E-EL-H0105c, brand: Elabscience; NF-κB Cat No. EK-H10890, brand: Ek- Bioscience). All procedures were performed according to the manufactures’ book.

### Statistical analysis

Statistical analyses were performed by SPSS 22.0 (SPSS Inc, Chicago, IL). Continuous data were denoted by mean ± standard deviation (SD) and analyzed by *t*-test or analysis of variance. Categorical data were denoted by percent (%) and analyzed using chi-square test. Heredity equilibrium was assessed by Hardy–Weinberg test. Demographic variables between patients and controls were compared by the chi-square test and Student’s *t*-test. Comparisons of genotype and allele frequencies between the case and control groups were evaluated by chi-square test. The genotype relative risk was calculated using the odds ratio (OR) and a 95% confidence interval (95%CI).The Kaplan–Meier method was performed for survival analysis and log-rank test was calculated. A multivariate Cox-proportional hazard model adjusting age, gender, body mass index (BMI), history of smoking and alcohol consumption. Progressive stepwise likelihood ratio was used to estimate the adjusted OR and 95%CI. All *P* values were double tailed and *P<0.05 was considered statistically significant.

## Results

### Characterization of study population

The characterization of study population was shown in Table 2. The case group consisted of 210 ARDS patients, with 107 males and 103 females aged between 22 and 73 years old. The control group enrolled 210 individuals who were identified as at-risk patients but did not meet criteria for ARDS, 103 males and 107 females with the mean age of 57.4 ± 9.1 years. The baseline characteristics of the two groups indicated that there was no statistically significant differences in age, sex composition, BMI, family history of tumor, smoking and alcohol status (*P*>0.05).

**Table 2:** Comparisons of baseline characteristics between the case group and the control group

Characteristics	Case group (*n*=210)	Control group (*n*=210)	*P* value
Age (year, mean ± SD)	58.4 ± 8.9	57.4 ± 9.1	0.256
Sex (*n*, %)			0.696
Male	107 (51.0%)	103 (49.0%)	
Female	103 (49.0%)	107 (51.0%)	
BMI (kg/m^2^, mean ± SD)	22.3 ± 1.7	22.5 ± 1.8	0.242
Smoking history (*n*, %)			0.470
Yes	67 (31.9%)	74 (35.2%)	
No	143 (68.1%)	136 (64.8%)	
Alcohol history (*n*, %)			0.545
Yes	75 (35.7%)	81 (38.6%)	
No	135 (64.3%)	129 (61.4%)	

Abbreviation: BMI, body mass index.

### Association between these SNPs and ARDS susceptibility

The genotype and allele frequencies of three SNPs (rs763780, rs2275913, rs8193036) of IL-17 and rs3774934 of NF-κB1 between the case and control groups were summarized in Table 3. The genotype distributions of all our studied loci were ascertained in a balanced state in the Chinese population based on Hardy–Weinberg equilibrium (*P*>0.05). No association was found between IL-17 rs763780 and NF-κB1 rs3774934 with ARDS risk, as calculated by both the dominant model and recessive model (*P*>0.05). A significantly decreased risk of ARDS was found in individuals carrying the mutant A-allele of rs2275913 at IL-17 (*P*<0.05), and this risk was significantly increased in individuals carrying the mutant T-allele of IL-17 rs8193036.

**Table 3:** Distribution of IL-17 rs763780, rs2275913, rs8193036 and NF-κB1 rs3774934 polymorphisms and ARDS risk

SNP	Case group (*n*=210)	Control group (*n*=210)	*P* value	OR (95%CI)	*P* value*	OR (95%CI)*
IL-17 rs763780						
Genotype						
AA	180 (85.7%)	185 (88.1%)	Ref			
AG	18 (8.6%)	20 (9.5%)	0.819	0.925 (0.451–1.896)	0.954	0.961 (0.626–1.329)
GG	12 (5.7%)	5 (2.4%)	0.086	2.467 (0.787–8.206)	0.142	1.431 (0.882–1.826)
AA+AG	198 (94.3%)	205 (97.6%)	Ref			
GG	12 (5.7%)	5 (2.4%)	0.083	2.485 (0.794–8.247)	0.137	1.437 (0.886–1.830)
AA	180 (85.7%)	185 (88.1%)	Ref			
AG+GG	30 (14.3%)	25 (11.9%)	0.470	1.233 (0.673–2.263)	0.563	1.106 (0.809–1.415)
Allele						
A	378 (90.0%)	390 (92.9%)	Ref			
G	42 (10.0%)	30 (7.1%)	0.139	1.444 (0.862–2.425)	0.175	1.185 (0.927–1.438)
IL-17 rs2275913						
Genotype						
GG	158 (75.2%)	89 (42.4%)	Ref			
GA	35 (16.7%)	45 (21.4%)	0.001	0.438 (0.254–0.754)	0.002	0.684 (0.509–0.887)
AA	17 (8.1%)	76 (36.2%)	<0.001	0.126 (0.067–0.234)	<0.001	0.286 (0.176–0.439)
GG+GA	193 (91.9%)	134 (63.8%)	Ref			
AA	17 (8.1%)	76 (36.2%)	<0.001	0.155 (0.084–0.283)	<0.001	0.310 (0.190–0.477)
GG	158 (75.2%)	89 (42.4%)	Ref			
GA+AA	52 (24.8%)	121 (57.6%)	<0.001	0.242 (0.156–0.375)	<0.001	0.470 (0.364–0.599)
Allele						
G	351 (83.6%)	223 (53.1%)	Ref			
A	69 (16.4%)	197 (46.9%)	<0.001	0.223 (0.159–0.311)	<0.001	0.424 (0.340–0.523)
IL-17 rs8193036						
Genotype						
CC	74 (35.2%)	137 (65.2%)	Ref			
CT	109 (51.9%)	53 (25.2%)	<0.001	3.807 (2.414–6.017)	<0.001	1.919 (1.547–2.363)
TT	27 (12.9%)	20 (9.5%)	0.004	2.499 (1.254–5.001)	0.007	1.638 (1.143–2.186)
CC+CT	183 (87.%)	190 (90.5%)	Ref			
TT	27 (12.9%)	20 (9.5%)	0.279	1.402 (0.729–2.703)	0.353	1.171 (0.846–1.496)
CC	74 (35.2%)	137 (65.2%)	Ref			
CT+TT	136 (64.8%)	73 (34.8%)	<0.001	3.449 (2.265–5.260)	<0.001	1.855 (1.504–2.289)
Allele						
C	257 (61.2%)	327 (77.9%)	Ref			
T	163 (38.8%)	93 (22.1%)	<0.001	2.230 (1.629–3.054)	<0.001	1.447 (1.262–1.643)
NF-κB1 rs3774934						
Genotype						
GG	118 (56.2%)	104 (49.5%)	Ref			
GA	49 (23.3%)	49 (23.3%)	0.603	0.881 (0.533–1.458)	0.690	0.941 (0.726–1.189)
AA	43 (20.5%)	57 (27.1%)	0.092	0.665 (0.402–1.099)	0.117	0.809 (0.609–1.048)
GG+GA	167 (79.5%)	153 (72.9%)	Ref			
AA	43 (20.5%)	57 (27.1%)	0.109	0.691 (0.429–1.114)	0.136	0.824 (0.625–1.054)
GG	118 (56.2%)	104 (49.5%)	Ref			
GA+AA	92 (43.8%)	106 (50.5%)	0.171	0.765 (0.511–1.144)	0.204	0.874 (0.713–1.069)
Allele						
G	285	257	Ref			
A	135	163	0.043	0.747 (0.557–1.002)	0.052	0.862 (0.737–1.001)

Notes: “OR*” adjusted by age, sex, alcohol and smoking; CI, confidence interval.

### Association between these SNPs and 30-day survival of ARDS

**Figure 2:**
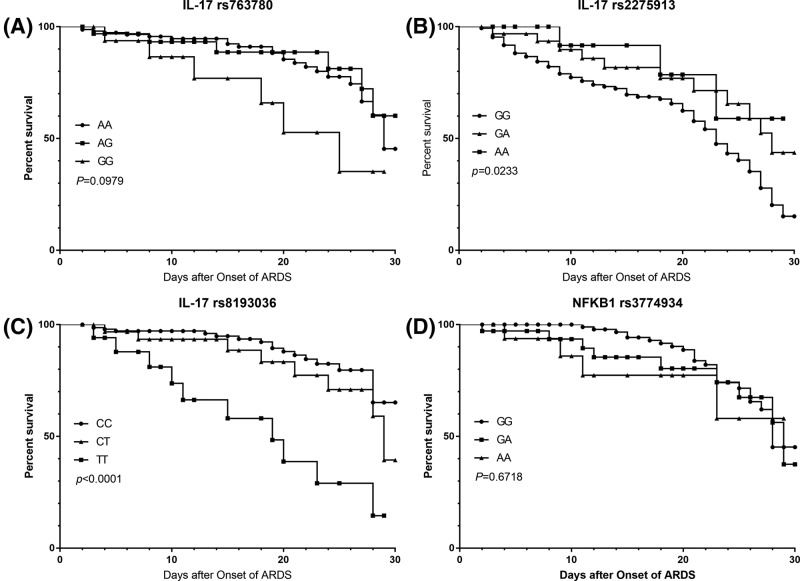
Kaplan–Meier plots of 30-day survival curves Kaplan–Meier plots of 30-day survival curves according to (**A**) IL-17 rs763780, (**B**) IL-17 rs2275913, (**C**) IL-17 rs8193036 and (**D**) NF-κB1 rs3774934 genotypes.

The impact of these SNPs on 30-day mortality of ARDS patients was shown in Figure 2. No association was identified between IL-17 rs763780 SNP and 30-day mortality of ARDS (*P*=0.097). Similarly, there was no association found between SNP of NF-κB1 rs3774934 and 30-day mortality (*P*=0.672). The homozygote AA genotype and heterozygote GA genotypes of IL-17 rs2275913 possessed an increased 30-day survival compared with the wild-type GG genotype (*P*=0.023). For IL-17 rs8193036 SNP, individuals with the homozygote TT and heterozygote CT genotypes had shorter 30-day survival time (*P*<0.05).

### Effect of these SNPs on serum IL-17 and NF-κB1 level

**Figure 3:**
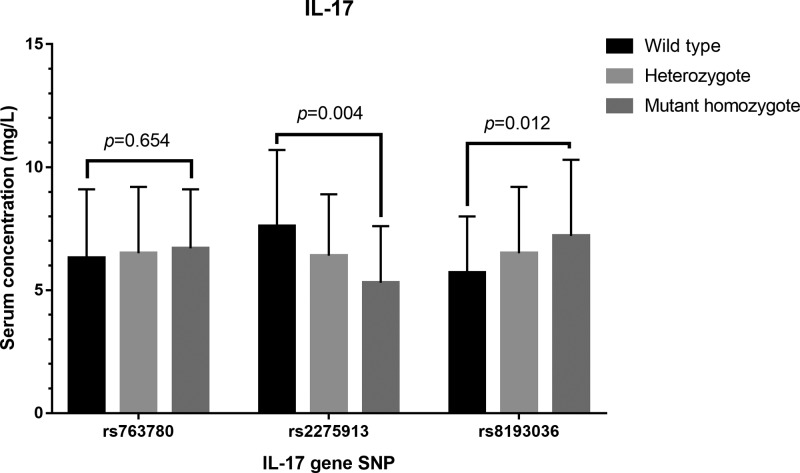
Plasma levels of IL-17 Plasma levels of IL-17 with the allelic distribution of IL-17 variants, including rs763780, rs2275913 and rs8193036 genotypes.

**Figure 4:**
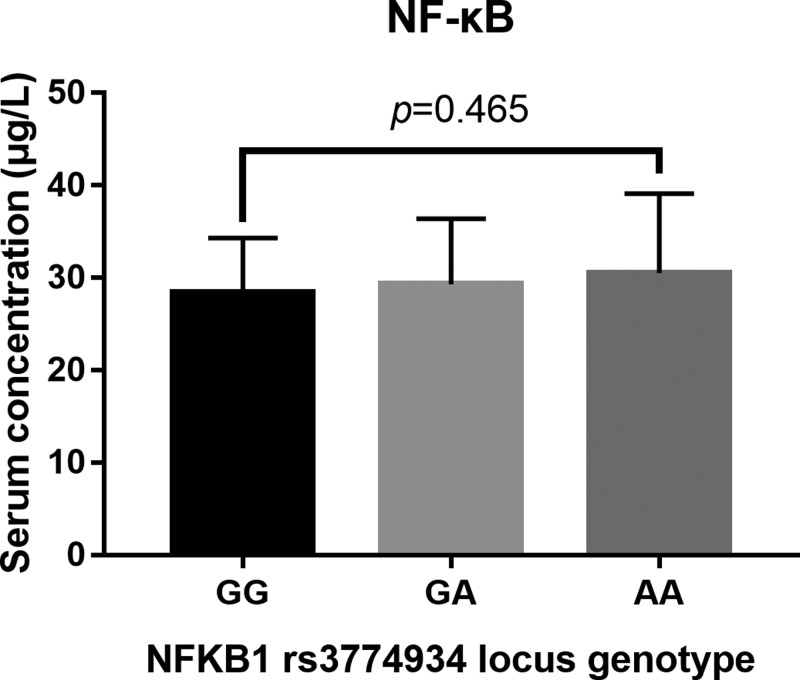
Plasma levels of NF-κB1 Plasma levels of NF-κB1with the allelic distribution of NF-κB1 rs3774934 variants.

We further evaluated whether these gene polymorphisms could affect serum IL-17 and NF-κB1 levels. As shown in Figure 3, no association was identified between SNP of IL-17 rs763780 and serum IL-17 level (*P*=0.654). Compared with individuals carrying the wild-type GG genotype, the AA-homozygous and GA-heterozygous carriers were found to have significantly decreased levels of IL-17 (*P*=0.004). T-allele carriers of rs8193036 at IL-17 gene, including individuals with the homozygote TT and heterozygote CT genotypes had significantly increased levels of IL-17 (*P*=0.012). There was no relationship between the levels of NF-κB1 and NF-κB1 rs3774934 genetic variants (*P*>0.05) (Figure 4).

## Discussion

To our knowledge this is the so far first study that estimated the association of IL-17 (rs763780, rs2275913 and rs8193036) and NF-κB1 (rs3774934) polymorphisms with ARDS susceptibility and prognosis. In the present study, we found two functional polymorphisms of IL-17, rs2275913 and rs8193036 were associated with ARDS risk and prognosis, indicating that the two genetic variants might act as possible markers for the prediction of ARDS risk and development.

ARDS is a clinical syndrome with heterogeneous etiologic factors and complicate pathogenesis [14]. The exact mechanisms underlying the pathogenesis of ARDS remains unclear; however, it is generally accepted that ARDS is a form of inflammatory disease and the immune regulation disorder may be an important factor in initiating inflammation [15–17]. As a proinflammatory cytokine, IL-17 has gained much attention. IL-17 family contains many expression forms, including IL-17A-F, among which IL-17A can extensively activate inflammation response and has been shown to be involved in a series of inflammatory diseases [18,19]. IL-17A abnormally increased in bronchial lavage fluid from the very early time of ALI, suggesting that disruption of IL-17 might contribute in the early phases of ARDS development and progression [20]. Besides, elevated circulating and alveolar levels of IL-17A were found to be associated with increased percentage of alveolar neutrophils, alveolar permeability, and organ dysfunction in ARDS [21]. Gene polymorphisms of IL-17A, such as rs2275913 and rs8193036, were reported to affect the development of many inflammatory diseases. SNPs of rs2275913 and rs8193036 are located in IL-17A gene, which were associated with ARDS risk and outcome. Individuals carrying the mutant A-allele of rs2275913 had decreased ARDS risk and better 30-day survival outcome, whereas individuals with the mutant T-allele off rs8193036 had otherwise increased ARDS susceptibility and shorter 30-day survival, indicating that the former mutant A-allele served a protective role while the latter mutant T-allele served a pathogenic one. The rs763780 is located in IL-17F gene. SNP of rs763780 had no impact on the risk and prognosis of ARDS (*P*>0.05). This observation was consistent with the previous finding that the A>G at rs763780 did not cause amino acid substitution and had no impact on IL-17 expression [22].

We further investigated whether SNPs of IL-17 can affect serum levels of IL-17. Data revealed that the mutant A-allele carriers of rs2275913 had decreased serum concentration of IL-17, while the mutant T-allele carriers of rs8193036 had increased concentration of serum IL-17, suggesting that rs2275913 and rs8193036 were functional polymorphisms that can affect IL-17 expression. Located in the regulatory domain of IL-17, SNP of rs2275913 can reduce IL-17 expression, relieve severe inflammatory reaction and intervene ARDS development [23]. Nevertheless, C>T at rs8193036 can increase IL-17 expression, activate and boost the inflammatory reaction, which finally contribute to higher risk of ARDS [24]. The distribution profiles of the two SNPs were consistent with that on ARDS risk and prognosis, indicating that rs2275913 and rs8193036 should affect ARDS initiation and progression via regulating the expression of IL-17. Further studies were needed to elucidate the underlying biological mechanisms leading to the association of SNPs and IL-17 expression with the progression of ARDS susceptibility and prognosis. There was no association identified between the risk and prognosis of ARDS and SNP of NF-κB1 rs3774934 genetic variants. Also, this SNP did not have significant impact on the plasma level of NF-κB1. From this perspective, SNP of rs3774934 at NF-κB1 gene was not involved in ARDS initiation and development.

Several limitations should be considered while interpreting the result of the present study. First, this first limitation was that the present study might be not powered to detect an association with a relatively modest sample and effect size, particularly for variants with low minor frequencies. Second, ARDS is a syndrome with heterogeneous causes and likely with heterogeneity with respect to mechanistic pathways. We cannot exclude the possibility that gene polymorphisms for the pathways of interest might have effects that may only be identified based on analysis of specific subsets of patients. Third, the effects of race and geographical distribution were not taken into consideration. Validation by a larger study is needed to confirm these findings.

## Conclusion

The presented study suggested that two functional polymorphisms of IL-17, including rs2275913 and rs8193036 were associated with ARDS risk and prognosis, indicating that IL-17 polymorphisms might act as possible markers for the prediction of ARDS risk and development. Validation by a larger prospective study from a more diverse ethnic population is needed to confirm these findings.

## Abbreviations

ALI: acute lung injury
ARDS: acute respiratory distress syndrome
IL-17: interleukin-17
NF-κB: nuclear factor κB
SNP: single nucleotide polymorphism
